# Variability and cost implications of three generations of the Roche LightCycler® 480

**DOI:** 10.1371/journal.pone.0190847

**Published:** 2018-01-12

**Authors:** Maria Dullaert-de Boer, Onno W. Akkerman, Marloes Vermeer, Dorine L. J. Hess, Huib A. M. Kerstjens, Richard M. Anthony, Tjip S. van der Werf, Dick van Soolingen, Adri G. M. van der Zanden

**Affiliations:** 1 Laboratory for Medical Microbiology and Public Health, Hengelo, The Netherlands; 2 University of Groningen, University Medical Center Groningen, Department of Pulmonary diseases and Tuberculosis, Groningen, The Netherlands; 3 ZGT Academy, ZGT, Almelo The Netherlands; 4 Tuberculosis reference laboratory, Center for Infectious Disease Research, Diagnostics and Perinatal Screening (IDS), National Institute for Public Health and the Environment (RIVM), Bilthoven, The Netherlands; 5 University of Groningen, University Medical Center Groningen, Department of Internal Medicine, Groningen, The Netherlands; University of Helsinki, FINLAND

## Abstract

Real time PCR has become a dominant method for the highly sensitive detection of pathogens in clinical material. Real time PCR can generate a fluorescence signal by using fluorescence labelled probes, allowing us to detect and semi quantify the amount of amplified DNA. Here we test the variability of the detection system and cost implications of three different versions of the LightCycler® 480 (LC480), focusing on the intensity of fluorescence and Cq in monoplex and multiplex rtPCRs.

For gastro-intestinal pathogens there was no correlation between the intensity of fluorescence and the Cq value in the different LC480 types. For probes with the dyes FAM^TM^, HEX^TM^, Cy5 and Red610 a higher fluorescence intensity was seen in LC480 type II and III compared to LC480 type I. After lowering the probe concentration for the Cy5 dye three-fold (from 0.3μM to 0.1μM) the Cq value remains the same and the intensity of fluorescence decreases. For the LC480 type II and III the difference in fluorescence intensity was much more extreme. The concentration of the different labelled probes can be lowered at least six-fold in LC480 type II and III cyclers while maintaining a fluorescence intensity as high as achieved in the LC480 type I with undiluted probe. In conclusion, the strength of the fluorescence signal of the LightCycler® 480 type III is superior to that of LightCycler® 480 types I and II, allowing the use of lower probe concentrations for all dyes, particularly for the dyes Red610 and Cy5. This results in a two thirds reduction in PCR probe costs. Switching to these newer machines for real-time PCR can reduce dye labelled probe consumption and thus reduce costs significantly.

## Introduction

Real time PCR (rtPCR) has become a widespread diagnostic tool in microbiology [1–2] allowing the highly sensitive detection of many different pathogens [3]. A monoplex rtPCR contains one specific primer pair and a probe and specifically amplifies a DNA sequence, specific for a single pathogen. A multiplex rtPCR contains several pairs of specific primers and probes in one rtPCR mixture, specific for several sequences of microorganisms. Multiplex and monoplex rtPCR assays are able to detect bacteria, viruses and or parasites [4–6].

In order to detect and semi quantify the amount of amplified DNA, the rtPCR assay needs to generate a measurable signal. Currently, most assays use fluorescent dyes to directly detect the PCR amplicons avoiding the need for post amplification manipulation [7–8]. A series of DNA probes each specific for a micro-organism, labelled with different fluorescence dyes, such as FAM^TM^, HEX^TM^, Red610 or Cy5 can be used simultaneously to signal the presence of specific targets resolved by instrument filters, allowing the detection of the PCR products after 20 to 45 cycles [9–10].

The quantification cycle (Cq) is proportional to the amount of DNA present in the clinical sample examined. The threshold for detecting fluorescence is a fluorescence intensity above the baseline that can be considered significantly above the background. Using Roche’s LightCycler® 480, the baseline is termed the “noise band”. Using the “noise band” option the standard deviation of the background signals (noise) of all samples included in the run is calculated. The noise band is then set to 12-fold of this standard deviation. Once the background noise has been removed, a log-line is calculated for each amplification curve and extrapolated from the threshold line as mentioned in the Roche Instrument operator’s manual, software version 1.5. The quantification performance of Roche’s LightCycler® 480 is indicated by Crossing point (Cp) and will be represented as Cq [11].

Recently, the LightCycler® 480 type I was succeeded by the LightCycler® 480 type II. The latter system differs from the first version in the block cycler units and the detection unit that contains the lamp unit and optics unit. LightCycler® 480 types I and II have a lamp unit containing a Xenon lamp. Whereas the LightCycler® type III has a LED lamp in its lamp unit, but produces the same spectra as LightCycler® 480 type II (Table 1).

**Table 1:** Primers and probes used.

Gastro-intestinal multiplex rtPCRs			
Pathogen target	Primers /probe	Primers/ probe 5’→3’	reference
*Salmonella species*	SE-ttr-6FSE-ttr-4RSE-ttr-5TP	CTC ACC AGG AGA TTA CAA CAT GGAGC TCA GAC CAA AAG TGA CCA TCCAC CGA CGG CGA GAC CGA CTT T	^[^^12^^]^
*Campylobacter jejuni*	CJ-mapA-F CJ-mapA-R CJ-mapA-MGB	CTG GTG GTT TTG AAG CAA AGA TTCAA TAC CAG TGT CTA AAG TGC GTT TATAAT TCC AAC ATC GCT AAT G	^[^^13^^]^
*Campylobacter coli*	Cc-ceuE-fw Cc-ceuE4-re Cc-ceuE-pr-FAM	AAG CTC TTA TTG TTC TAA CCA ATT CTA ACATCC ATG TGT GCC TAC TTT TAC ATTTTG GAC CTC AAT CTC GCT TTG GAA TCA TT	^[^^14^^]^
*Shigella dysenteriae* / Enteroinvasive *Escherichia coli* (EIEC)	IpaH-U1IpaH-L1IpaH-TMIpaH-TM-610	CCT TTT CCG CGT TCC TTG ACGG AAT CCG GAG GTA TTG CCGC CTT TCC GAT ACC GTC TCT GCACGC CTT TCC GAT ACC GTC TCT GCA	^[^^14^^]^
*Yersinia enterocolitica*	ystB_FystB_Rail-PystB_PYe-ail-fw1Ye-ail-fw3Ye-ail-re	TAG CCG CTG AGA TAA ACA GAA AAGCAT CAT TTT CTT CTG AAG GCG ACATAAAGGCTAACATATTCTGCGATACTCAGACCCGGGCCATCTTTCCGCATTAGGGCCATCTTTCCGCATCCGTATGCCATTGACGTCTTACT	^[^^15^^–^^17^^]^
Shiga-toxin producing *Escherichia coli* (STEC)	Stx1F934-modStx1F934F-mod1dStx2F-LvIStx1R1042-GStx1R1042-modCStx1R1042-mod1dStx2R-G-LvIStx2R-A-LvIStx1P990-mod-MGBStx1P990-mod1c-MGBStx1P990-mod1d-MGBStx2P-LvI-MGBStx2F-mod4-SLEStx2R-mod4-SLEStx2P-mod3-SLE	TGG CAT TAA TAC TGA ATT GTC ATC ATCTGG CAT TAA TAT TAA ATT GCC ATC ATCCG GAA TGC AAA TCA GTC GTGCG TAA TCC CAC GGA CTC TTCGCG TAA TCC CAC GCA CTC TTGAG TAA TCC CAC GCC CAC TTCACC ACT GAA CTC CAT TAA CGC CTAC CAC TAA ACT CCA TTA ACG CCATTC CTT CTA TGT GTC CGG CAGCCT TCT ATG TGC CCG GTA GTCC TTC TAT GTG CCC GAC AGACT CAC TGG TTT CAT CAT ACAG GAT CTT ACT GAA CCA AAC CAA TCAT CCT CAT TAT ACT TGG AAA ACT CAA TTCCA TGG CGG CGG ATT GTG C	^[^^18^^]^
*Giardia lamblia*	TM-Giardia-80FTM-Giardia-127RTM-Giardia-105	GAC GGC TCA GGA CAA CGG TTTTG CCA GCG GTG TCC GCCC GCG GCG GTC CCT GCT AG	^[^^19^^]^
*Cryptosporidium species*	TM-Crypto-fwTM-Crypto-reCrypto-pr-610	CGCTTCTCTAGCCTTTCATGACTTCACGTGTGTTTGCCAATCCAATCACAGAATCATCAGAATCGACTGGTATC	^[^^19^^]^
*Dientamoeba fragillis*	Df-124-fw Df-221-reDf-172-pr-vic	CAACGGATGTCTTGGCTCTTTATGCATTCAAAGATCGAACTTATCACCAATTCTAGCCGCTTAT	^[^^20^^]^

The aim of this study was to test the variability of the detection system, especially the differences in illumination and thus intensity of the fluorescence produced, and implications for running costs when using the different LightCycler® 480 (LC480) models to run multiplex rtPCRs. This is highly relevant for most Real-time PCR machines, and in case for the LC480 models as most laboratories worldwide still use type I and II, while the most up-to-date have already switched to type III. Many large volume laboratories utilize different versions of PCR machinery from one manufacturer, so cross comparability is also critically important.

## Methods

### LightCycler® 480 instruments

Our laboratory utilizes three types of Roche LC480 Instruments. Type I, type II and one modified type II which utilise a LED lamp, referred to as a type III. All three types of Roche LC480 instruments were used according to the manufacturer’s instructions [Roche diagnostics Nederland BV]. All LightCyclers contain the same block cycler and same detection unit including the optical filters but differ in their lamp units.

### rtPCR assays

Four multiplex rtPCRs with different fluorescence dyes were used to study the performance of the detection systems (Tables 2 and 3). The total reaction volume of the multiplex rtPCR consisted of 20μL reaction mix and 10 μL of DNA extract. The reaction mix contained 3 μL of bovine serum albumin (20 mg/mL; Invitrogen, Breda, The Netherlands) which was added to 15 μL of Roche Probes Master (Roche Diagnostics Nederland BV, Almere, the Netherlands) and Molecular Grade Water (Roche Diagnostics Nederland BV, Almere, the Netherlands), primers and probes to bring the total reaction volume to 20 μL. These rtPCRs are routinely used for the molecular detection of gastro-intestinal pathogens. For each target a specific positive control is used. The four different multiplex rtPCRs were Molecular faeces panels 1 to 4 (MFP1 to 4). MFP1 detects *Salmonella species*, *Campylobacter jejuni and Campylobacter coli*, MFP2 detects *Shigella dysenteriae /* Enteroinvasive *Escherichia coli* EIEC and *Yersinia enterocolitica*. MFP3 detects *Shiga-toxin producing Escherichia coli* (STEC). MFP4 detects *Giardia lamblia*, *Cryptosporidium species*, *and Dientamoeba fragilis*. *Synechococcus*, detected with a probe labelled with a Cy5 dye, was used as the internal control (IC) and included in each of the multiplex rtPCRs MFP1, MFP2, MFP3 and MFP4. Cq values were recorded for each analysis, and the assay result was called positive or negative.

**Table 2:** Detection dyes with different excitation and emission spectra are shown for the LightCycler® 480 type I, II and III.

LightCycler® 480 type I			LightCycler 480® type II and type III		
Dye	λ Excitation filter (nm)	λ Emission filter (nm)	Dye	λ Excitation filter (nm)	λ Emission filter (nm)
FAM^TM^	450	533	FAM	440	510
HEX^TM^/VIC	483	568	HEX/VIC	465	580
Red610	523	610	Red610	498	610
Cy5	558	640	Cy5	533	640

**Table 3:** Pathogen targets of the gastro-enteritis multiplex rtPCRs with accompanying dyes and probe concentration used in the final rtPCR reaction.

Gastro-intestinal multiplex rtPCRs					
Pathogen Panel	Pathogen target	Primers / probe	manufacturer	label	Probe concentration μM
*MFP1*	*Salmonella enterocolitica*	SE-ttr-5TP	TIB*	Red610	0,15μM
	*Campylobacter jejuni*	CJ-mapA-MGB	LIFE**	HEX^TM^	0,15μM
	*Campylobacter coli*	Cc-ceuE-pr-FAM	TIB*	FAM^TM^	0,20 μM
	*Synechococcus*	cyano-pr-670	TIB*	Cy5	0,20μM
MFP2	Shigella dysenteriae / Enteroinvasive Escherichia coli (EIEC)	ipaH-TM-610	TIB*	Red610	0,15μM
	Yersinia enterocolitica	ail-P	LIFE**	HEX^TM^	0,15μM
		ystB_P	LIFE**	HEX^TM^	0,20μM
	*Synechococcus*	cyano-pr-670	TIB*	Cy5	0,20μM
MFP3	Shiga-toxin producing Escherichia coli (STEC)	Stx1P990-mod-MGB	LIFE**	FAM^TM^	0,10μM
		Stx1P990-mod1c-MGB	LIFE**	FAM^TM^	0,10μM
		Stx1P990-mod1d-MGB	LIFE**	FAM^TM^	0,10μM
		Stx2P-LvI-MGB	LIFE**	FAM^TM^	0,10μM
		Stx2P-mod3-SLE	TIB*	FAM^TM^	0,10μM
	*Synechococcus*	cyano-pr-670	TIB*	Cy5	0,20μM
MFP4	Giardia lamblia	TM-Giardia-105	TIB*	FAM^TM^	0,20μM
	Cryptosporidium species	Crypto-pr-610	TIB*	Red610	0,15μM
	Dientamoeba fragillis	Df-172-pr-vic	LIFE**	HEX^TM^	0,15μM
	*Synechococcus*	cyano-pr-670	TIB*	Cy5	0,20μM

* TIB MOLBIOL, Berlin, Germany

** Applied Biosystems^TM^ by Life technologies^TM^, Applied Biosystems UK, Renfrewshire, United Kingdom

### LightCycler®480 cycle conditions

Reaction conditions for all rtPCRs were 10 minutes Taq hotstart activation at 95°C, followed by 45 cycles of 95°C for 15 seconds and 60°C for 1 minute. The final step comprised cooling to 40°C for 20 seconds.

### Detection dyes

The dyes used were FAM^TM^, HEX^TM^/VIC, Red610 and Cy5.

### Intensity of fluorescence

The intensity of fluorescence were measured in units relative to the positive controls of the rtPCR assays.

### Statistical analysis

The results of the positive controls (DNA of laboratory strains) of the different gastro-intestinal rtPCR assays were collected over eight months. Calculations were made over two months during which the positive controls for the gastro-intestinal pathogens were tested with the same rtPCR reagents (mastermix batch number, primer probe batches, including the same batch of positive controls) and under the same specific conditions for the rtPCR. In those two months per LC480**®** ten results of each positive control in the gastro intestinal rtPCR were analysed using the same reagents and rtPCR conditions. Cp values were calculated using Roche Lightcycler software analyzing with LC480 Abs Quant/2nd Derivative Max. Continuous data are presented as means with standard deviation. Bar charts with standard error bars were used to visualize the fluorescence data. Statistical analysis was performed using the Statistical Package for the Social Sciences version 24 (SPSS Inc., Chicago, USA).

## Results

The three different types of Roche’s LC480 Instruments were compared regarding the intensity of fluorescence produced with identical positive controls. No correlation was seen between the fluorescence intensity and the Cq value (Table 4 and S1 Dataset).

**Table 4:** Relation of the Cq value and the level of fluorescence in the three different types of Roche LightCycler® 480. The mean and standard deviation of ten analyses per target is showed by the Cq value and fluorescence in units.

		Cq-value			Fluorescence in units		
		Type I	Type II	Type III	Type I	Type II	Type III
Target	Dye	LC I	LC II	LC III	LC I	LC II	LC III
**C.coli**	**FAM**^**TM**^	30,32	30,49	30,44	24,84	22,58	32,26
**Std. Deviation**		0,18	0,29	0,18	1,68	2,39	2,12
**STEC**		31,43	31,42	30,86	23,56	19,52	34,52
**Std. Deviation**		0,32	0,34	0,37	2,53	1,81	3,87
**G.lamblia**		31,82	31,81	31,81	19,32	18,20	25,26
**Std. Deviation**		0,11	0,76	0,89	1,30	1,13	1,94
**C.jejuni**	**HEX**^**TM**^	33,75	33,85	33,9	26,18	30,40	40,51
**Std. Deviation**		0,18	0,39	0,37	0,71	2,15	1,79
**Y.enterocolitica**		32,85	32,95	32,72	11,18	13,20	17,86
**Std. Deviation**		0,26	0,29	0,48	1,14	0,97	2,83
**D.fragilis**		31,90	31,83	31,65	21,60	23,52	34,10
**Std. Deviation**		0,20	1,05	0,34	1,82	2,39	1,09
**S.enterica**	**Red610**	31,06	31,08	31,10	6,80	12,42	19,83
**Std. Deviation**		0,18	0,43	0,32	0,53	1,35	2,87
**S.dysenteriae/EIEC**		33,42	33,47	33,17	6,66	11,21	18,58
**Std. Deviation**		0,33	0,28	0,61	0,36	1,15	1,52
**Cryptosporidium spp.**		34,48	34,15	33,98	4,66	8,36	13,46
**Std. Deviation**		0,31	0,36	2,63	0,36	0,50	1,23
**MFP1**	**CY5**	32,21	32,25	32,32	1,54	6,42	8,52
**Std. Deviation**		0,24	0,25	0,21	0,08	0,34	0,67
**MFP2**		32,21	32,31	32,25	1,42	6,12	8,42
**Std. Deviation**		0,23	0,20	0,22	0,17	0,26	1,04
**MFP3**		32,11	32,15	32,18	1,54	6,02	9,42
**Std. Deviation**		0,26	0,35	0,36	0,11	0,36	1,06
**MFP4**		32,21	32,32	31,98	1,62	6,94	9,40
**Std. Deviation**		0,15	0,81	0,64	0,12	0,41	1,60

**Fig 1:**
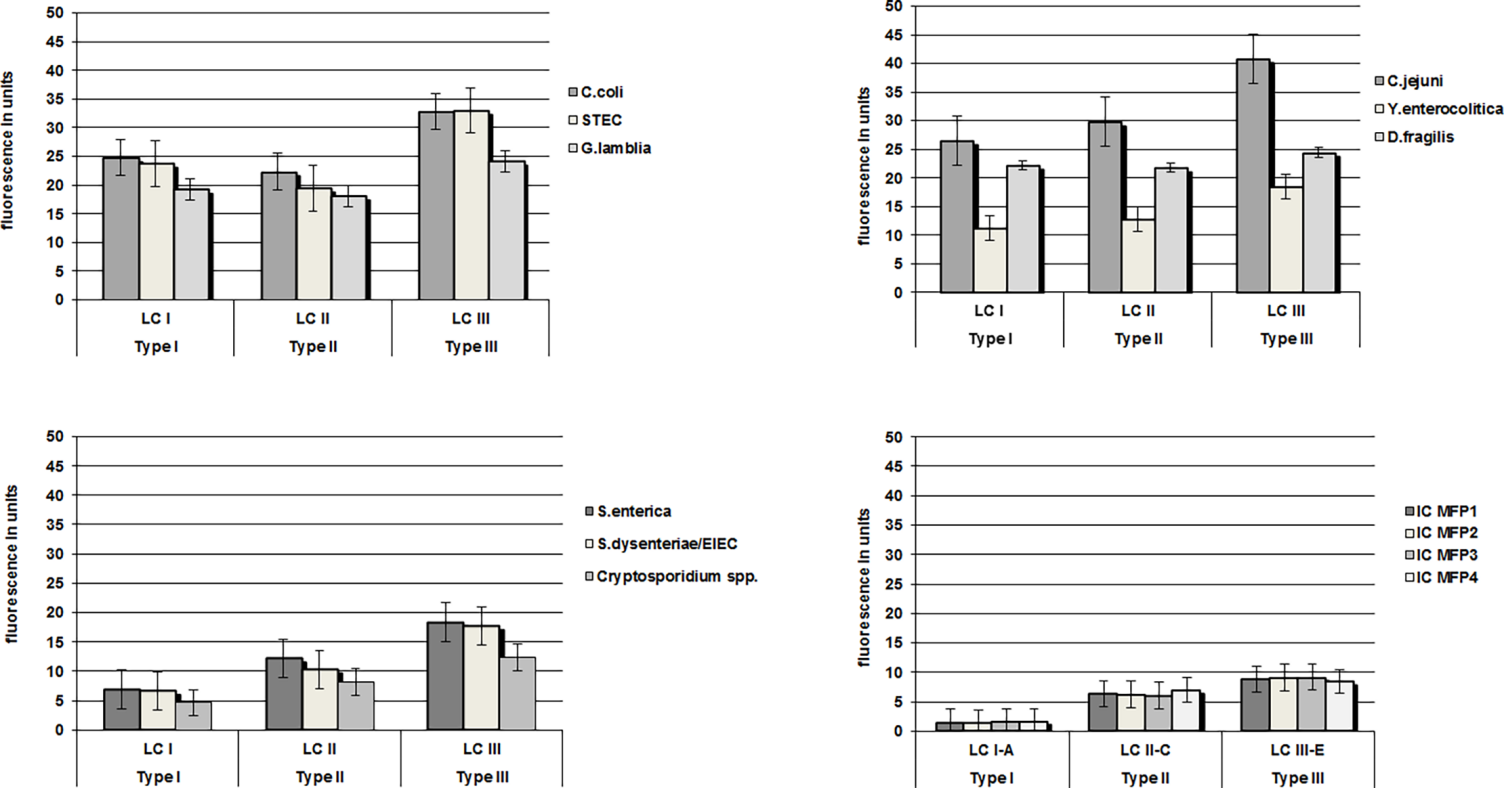
Fluorescence intensity in units of gastro-intestinal multiplex rtPCRs (A, B, C, D), measured by LightCycler® 480 instruments type I, II and III. The mean of ten analyses per target is showed by fluorescence in units. The X-axis represents the types of the PCR instrument and the Y-axis represents the fluorescence intensity in units. MFP: molecular fecal panel. *Synechococcus* is used as internal control (IC) of the targets and was detected with a probe labelled with a Cy5 dye included in the multiplex rtPCRs MFP1, MFP2, MFP3 and MFP4.

Although the Cq value of the specific positive controls did not differ between the three types of LC480 instruments, the absolute fluorescence intensity revealed an enormous range for all dyes, especially for the Red610 and Cy5 dyes. LC480 I showed the lowest fluorescence intensity for the multiplex rtPCRs whereas LC480 III showed the highest fluorescence intensity (Fig 1A–1D and S1 Dataset) for FAM^TM^, HEX^TM^, Red610 and Cy5 dyes.

**Fig 2:**
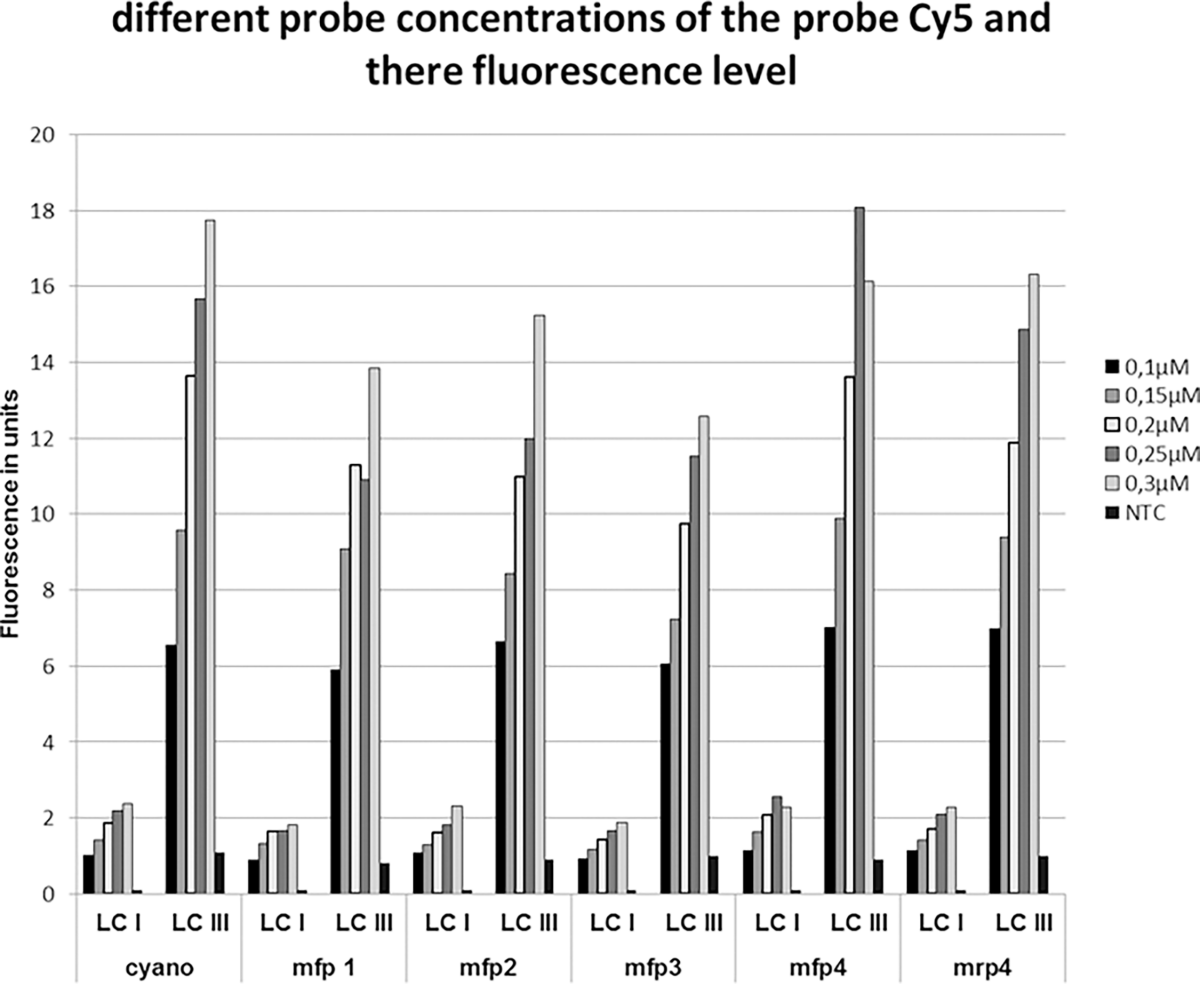
Correlation of the probe concentration and the intensity of fluorescence in two different types of Roche LightCycler® 480. The mean of five analyses per target is showed by fluorescence in units.

LC480 III is superior to LC480 I and II for the dyes FAM^TM^ and HEX^TM^. LC480 I and LC480 II differ a factor two in fluorescence intensity with LC480 III. The fluorescence intensity of Type LC480 I differed by a factor three to six for the dyes Red610 and Cy5 when compared to the Type LC480 II and Type LC480 III. The effect of changing the probe concentrations on the Cq value and the fluorescence intensity was studied for Type LCI and Type LCIII LightCyler480 as these two models had the most diverse fluorescence intensities. In different rtPCRs, the Cq value remained the same with an decrease of probe concentration from 0.3 μM to 0.1μM. By lowering the probe concentration in Type LCIII the fluorescence intensity remains the same or even higher in comparison to a Type LCI. This increase of the fluorescence level in Type LCIII is associated with a significant cost reduction for the dye Cy5 (Fig 2, S2 Dataset and Table 5).

**Table 5:** Reducing running costs by lowering the probe concentration in a LED Roche LightCycler® 480.

Gastro-intestinal multiplex rtPCRs						
Pathogen Panel	Pathogen target	Primers / probe	label	Costs 10000 rtPCR's	cost after reduction	
*MFP1*	*Salmonella enterocolitica*	SE-ttr-5TP	Red610	€ 1.209,84	1./3	€ 403,28
	*Campylobacter jejuni*	CJ-mapA-MGB	HEX^TM^	€ 658,12	1./2	€ 329,06
	*Campylobacter coli*	Cc-ceuE-pr-FAM	FAM^TM^	€ 764,99	1./2	€ 382,50
	*Synechococcus*	cyano-pr-670	Cy5	€ 764,99	1./6	€ 127,50
MFP2	Shigella dysenteriae / Enteroinvasive Escherichia coli (EIEC)	ipaH-TM-610	Red610	€ 1.209,84	1./3	€ 403,28
	Yersinia enterocolitica	ail-P	HEX^TM^	€ 679,08	1./2	€ 339,54
		ystB_P	HEX^TM^	€ 882,81	1./2	€ 441,41
	*Synechococcus*	cyano-pr-670	Cy5	€ 764,99	1./6	€ 127,50
MFP3	Shiga-toxin producing Escherichia coli (STEC)	Stx1P990-mod-MGB	FAM^TM^	€ 442,00	1./2	€ 221,00
		Stx1P990-mod1c-MGB	FAM^TM^	€ 442,00	1./2	€ 221,00
		Stx1P990-mod1d-MGB	FAM^TM^	€ 442,00	1./2	€ 221,00
		Stx2P-LvI-MGB	FAM^TM^	€ 442,00	1./2	€ 221,00
		Stx2P-mod3-SLE	FAM^TM^	€ 764,99	1./2	€ 382,50
	*Synechococcus*	cyano-pr-670	Cy5	€ 764,99	1./6	€ 127,50
MFP4	Giardia lamblia	TM-Giardia-105	FAM^TM^	€ 764,99	1./2	€ 382,50
	Cryptosporidium species	Crypto-pr-610	Red610	€ 1.209,84	1./3	€ 403,28
	Dientamoeba fragillis	Df-172-pr-vic	HEX^TM^	€ 704,85	1./2	€ 458,15
	*Synechococcus*	cyano-pr-670	Cy5	€ 764,99	1./2	€ 382,50
			Total	€ 13.677,31		€ 5.574,50

A six-fold difference in fluorescence intensity was seen between LC480 I and LC480 III demonstrating an increased sensitivity of the new LED lamp in the LC480 III for the Cy5 dye (Fig 2 and S2 Dataset). When the probe concentration was increased it results in a dramatic increase in the probe costs for the rtPCR (Table 5). Running cost could be reduced from 13677,31 euro to 5574,5 euro, a reduction of 8,102.81 euro (-59.2%) for 10,000 rtPCR's when only LC480 III is used for detection.

## Discussion

We assessed the Cq values and the intensity of fluorescence produced by three types of LC480 using 4 different multiplex rtPCRs. The LC480 type I and type II/ type III differ in their excitation spectra and this leads to variation in fluorescence intensity (Table 2). Overall, the lowest fluorescence for the dyes FAM^TM^, HEX^TM^, Red610 and Cy5 was detected by the LC480 type I instruments while the LC480 type III instrument detected the highest fluorescence intensity. For the FAM^TM^ and HEX^TM^ dyes two-fold variation in intensity was seen between the different versions of the LC480 instruments. Real time PCRs with dyes Red610 and Cy5 showed an enormous variation in fluorescence intensity between the three LC480 types tested. No relation between fluorescence intensity and Cq value was observed however.

The quant factor represents the maximum factor of fluorescence dynamics and is calculated by dividing the fluorescence at the plateau phase by the one at the background. The amplification conditions, detection system, lamp unit and software significantly influence the quant factor of an instrument. Theoretically, due to the variation in fluorescence output, positive results could be interpreted as negative if dye intensity levels used are too low. This is caused by an individual signal to noise ratio unique to each LC480 Instrument. Most of the assays have a default setting of the detection format, with a standard quant factor. To determine the relation between signal-to-noise ratio and the fluorescence intensity, the quant factor was changed from 10 to 1 and the probes were diluted one hundred times. The relation appeared linear; the signal-to-noise ratio remains the same in the detection at different concentrations of the labelled probes with different quant factor (S3 Dataset). This emphasizes the importance of optimal instrument settings of the LC480 to prevent false negative results.

Our laboratory has a comprehensive maintenance schedule for all PCR cyclers. Regular cleaning of the lens is needed as well as more extensive maintenance for all of the instruments to ensure optimal performance (S4 Dataset).

Fluorescence intensity is linear with respect to the number of copies of a probe until a plateau is reached after which no further increase in fluorescence is seen as the amount of probe increases. Each unique probe has its own signal level. Reagents used in rtPCR like the polymerase and primers, also influence detection limits [21–22]. There is also a difference in the fluorescence intensity achievable in monoplex and multiplex rtPCRs for *C*. *coli*, STEC and *Giardia*. This can be caused by the concentration of reaction mix components, such as primers, polymerase, master mix, magnesium, but also the analytical platforms, and cycling conditions as well as the characteristics of the probe [23–26]. Generally, as multiplex rtPCRs are more complex than monoplex rtPCRs, they are more sensitive to the initial reaction conditions, due to factors like competition between individual rtPCRs as well as increased probability of the formation of primer dimers [27].

For all real-time PCR machines, fluorescence intensity levels also depend on the maintenance, timely replacement of lamp units—and cleanliness of the instruments lens. These interventions will also allow a lower concentration of rtPCR labelled probes to produce a reliably measurable signal. This maintenance is often overlooked in routine laboratories or the verification of the expected fluorescence intensity is not performed, these interventions are not expensive but when performing large numbers of diagnostic rtPCRs labelled probes are a significant cost.

In conclusion, the fluorescence output of the LC480 type III is superior to that of LC480 types I and II for all dyes, especially the dyes Red610 and Cy5. Detection with a LED lamp enables lowering the concentration of a probe while maintaining an acceptable signal level, thereby reducing the running costs by 59.2%.

## Supporting information

S1 Dataset S1 Dataset-data statistical analyses Figure 1 and Table 4. (XLSX)

S2 Dataset S2 Dataset-figure 2 probe titratie LC comparison. (XLSX)

S3 Dataset S3 Dataset- data not shown line 243 signal to noise test. (XLS)

S4 Dataset S4 Dataset-data not shown line 248 all data maintenance. (XLSX)
