# Correction: Variability and cost implications of three generations of the Roche LightCycler® 480

**DOI:** 10.1371/journal.pone.0192946

**Published:** 2018-02-09

**Authors:** Maria Dullaert-de Boer, Onno W. Akkerman, Marloes Vermeer, Dorine L. J. Hess, Huib A. M. Kerstjens, Richard M. Anthony, Tjip S. van der Werf, Dick van Soolingen, Adri G. M. van der Zanden

In Table 1 the names and sequences of the primer and probes are formatted incorrectly. Please see the corrected Table 1 here.

**Table 1:** Primers and probes used.

Gastro-intestinal multiplex rtPCRs			
Pathogen target	Primers /probe	Primers/ probe 5’→3’	reference
*Salmonella species*	SE-ttr-6F	CTC ACC AGG AGA TTA CAA CAT GG	^[12]^
	SE-ttr-4R	AGC TCA GAC CAA AAG TGA CCA TC	
	SE-ttr-5TP	CAC CGA CGG CGA GAC CGA CTT T	
*Campylobacter jejuni*	CJ-mapA-F	CTG GTG GTT TTG AAG CAA AGA TT	^[13]^
	CJ-mapA-R	CAA TAC CAG TGT CTA AAG TGC GTT TAT	
	CJ-mapA-MGB	AAT TCC AAC ATC GCT AAT G	
*Campylobacter coli*	Cc-ceuE-fw	AAG CTC TTA TTG TTC TAA CCA ATT CTA ACA	^[14]^
	Cc-ceuE4-re	TCC ATG TGT GCC TAC TTT TAC ATT	
	Cc-ceuE-pr-FAM	TTG GAC CTC AAT CTC GCT TTG GAA TCA TT	
*Shigella dysenteriae* / Enteroinvasive *Escherichia coli* (EIEC)	IpaH-U1	CCT TTT CCG CGT TCC TTG A	^[14]^
	IpaH-L1	CGG AAT CCG GAG GTA TTG C	
	IpaH-TM	CGC CTT TCC GAT ACC GTC TCT GCA	
	ipaH-TM-610	CGC CTT TCC GAT ACC GTC TCT GCA	
*Yersinia enterocolitica*	ystB_F	TAG CCG CTG AGA TAA ACA GAA AAG	^[15–17]^
	ystB_R	CAT CAT TTT CTT CTG AAG GCG A	
	ail-P	CATAAAGGCTAACATATTC	
	ystB_P	TGCGATACTCAGACCC	
	Ye-ail-fw1	GGGCCATCTTTCCGCATTA	
	Ye-ail-fw3	GGGCCATCTTTCCGCAT	
	Ye-ail-re	CCGTATGCCATTGACGTCTTACT	
Shiga-toxin producing *Escherichia coli* (STEC)	Stx1F934-mod	TGG CAT TAA TAC TGA ATT GTC ATC ATC	^[18]^
	Stx1F934F-mod1d	TGG CAT TAA TAT TAA ATT GCC ATC AT	
	Stx2F-LvI	CCG GAA TGC AAA TCA GTC GT	
	Stx1R1042-G	GCG TAA TCC CAC GGA CTC TTC	
	Stx1R1042-modC	GCG TAA TCC CAC GCA CTC TT	
	Stx1R1042-mod1d	GAG TAA TCC CAC GCC CAC TTC	
	Stx2R-G-LvI	ACC ACT GAA CTC CAT TAA CGC C	
	Stx2R-A-LvI	T**A**C CAC TAA ACT CCA TTA ACG CCA	
	Stx1P990-mod-MGB	TTC CTT CTA TGT GTC CGG CAG	
	Stx1P990-mod1c-MGB	CCT TCT ATG TGC CCG GTA G	
	Stx1P990-mod1d-MGB	TCC TTC TAT GTG CCC GAC AG	
	Stx2P-LvI-MGB	ACT CAC TGG TTT CAT CAT A	
	Stx2F-mod4-SLE	CAG GAT CTT ACT GAA CCA AAC CAA T	
	Stx2R-mod4-SLE	CAT CCT CAT TAT ACT TGG AAA ACT CAA TT	
	Stx2P-mod3-SLE	CCA TGG CGG CGG ATT GTG C	
*Giardia lamblia*	TM-Giardia-80F	GAC GGC TCA GGA CAA CGG TT	^[19]^
	TM-Giardia-127R	TTG CCA GCG GTG TCC G	
	TM-Giardia-105	CCC GCG GCG GTC CCT GCT AG	
*Cryptosporidium species*	TM-Crypto-fw	CGCTTCTCTAGCCTTTCATGA	^[19]^
	TM-Crypto-re	CTTCACGTGTGTTTGCCAAT	
	Crypto-pr-610	CCAATCACAGAATCATCAGAATCGACTGGTATC	
*Dientamoeba fragillis*	Df-124-fw	CAACGGATGTCTTGGCTCTTTA	^[20]^
	Df-221-re	TGCATTCAAAGATCGAACTTATCAC	
	Df-172-pr-vic	CAATTCTAGCCGCTTAT	
